# Multifunctional substrate of Al alloy based on general hierarchical micro/nanostructures: superamphiphobicity and enhanced corrosion resistance

**DOI:** 10.1038/srep35940

**Published:** 2016-10-24

**Authors:** Xuewu Li, Tian Shi, Cong Liu, Qiaoxin Zhang, Xingjiu Huang

**Affiliations:** 1 School of Mechanical and Electronic Engineering, Wuhan University of Technology, 122 Luoshi Road, Wuhan 430070, P.R. China; 2 School of Machinery and Automation, Wuhan University of Science and Technology, 947 Peace Avenue, Wuhan 430081, P.R. China

## Abstract

Aluminum alloys are vulnerable to penetrating and peeling failures in seawater and preparing a barrier coating to isolate the substrate from corrosive medium is an effective anticorrosion method. Inspired by the lotus leaves effect, a wetting alloy surface with enhanced anticorrosion behavior has been prepared via etch, deposition, and low-surface-energy modification. Results indicate that excellent superamphiphobicity has been achieved after the modification of the constructed hierarchical labyrinth-like microstructures and dendritic nanostructures. The as-prepared surface is also found with good chemical stability and mechanical durability. Furthermore, superior anticorrosion behaviors of the resultant samples in seawater are investigated by electrochemical measurements. Due to trapped air in micro/nanostructures, the newly presented solid-air-liquid contacting interface can help to resist the seawater penetration by greatly reducing the interface interaction between corrosive ions and the superamphiphobic surface. Finally, an optimized two-layer perceptron artificial neural network is set up to model and predict the cause-and-effect relationship between preparation conditions and the anticorrosion parameters. This work provides a great potential to extend the applications of aluminum alloys especially in marine engineering fields.

In recent decades, aluminum alloys (Al alloys) have been widely applied in engineering fields due to excellent physical, chemical, and mechanical properties^1^,^2^. Especially for the field of naval architecture and ocean engineering, Al alloys are pervasively used for plate and shell components, welding components, vessel equipments, and some other structural parts^3^. However, the reactive chlorine ions abounding in seawater can erode the protective oxidation films on substrates leading to the penetrating and peeling failures^4^, which also severely restricts their service life and application fields. Hence, it is of great economic and realistic significance to conduct the research on the protection of Al alloys from corrosion in seawater.

In industry, the classical processes of protective coating^5^,^6^,^7^,^8^, thermomechanical treatment^9^,^10^,^11^, surface oxidization^12^,^13^,^14^, mechanical alloying^15^,^16^, and corrosion inhibitors^17^,^18^ have been applied to improve corrosion resistances of Al alloys. Besides, Boinovich *et al.* have reported the AlMg3 alloys surfaces with enhanced resistance to pitting corrosion in sodium chloride solutions by using the nanosecond laser treatment^19^. Rao *et al.* have applied the friction stir process to render Al-30Si alloys superior corrosion resistance^20^. However, coating techniques with heavy metal ions will contaminate the environment. Micro-arc oxidations under high voltage can cause safety hazards. Mechanical alloying will easily oxidize and pollute the samples. Laser process is usually costly and hard to control. Hence, it is still a big challenge for Al alloys to develop a simple, eco-friendly, and low-cost anticorrosion approach.

Recent years have seen some eco-friendly organic/inorganic nanocomposite films^21^, polyaniline coatings^22^, conversion coatings^23^, superhydrophobic films^24^,^25^, and self-assembled films^26^. Among them, superhydrophobic surfaces with special wettability^27^,^28^ can enlighten a method to resist corrosive ions penetration by minishing the interface interaction. Inspired by the lotus leaves structures, a wetting surface can be achieved by preparing special rough structures and low-surface-energy coatings. Recently, some physical and chemical methods have been reported to fabricate superhydrophobic Al alloys surfaces, such as the wire cutting^29^, laser processing^30^, numerically controlled milling^31^, electrolyte jet machining^32^, magnetron sputtering^33^, template replication^34^, anodic oxidation^35^, sol-gel^36^, etch^37^, *in situ* crystallization^38^, hydrothermal process^39^, and hybrid coatings^40^. However, few works report the corrosion resistances of prepared surfaces in corrosive seawater and oily mediums. And the chemical stability and mechanical durability for application of prepared surfaces are seldom investigated. Moreover, preparing a superoleophobic surface is more difficult than the superhydrophobic one for that it has to repel oils with lower surface tensions. Thus, more complex micro/nanostructures need to be prepared to render Al alloys superamphiphobic and anticorrosion behaviors.

Herein, a simple, eco-friendly, and low-cost method is developed to prepare multifunctional Al alloys surfaces. The superamphiphobic hierarchical bimetallic micro/nanostructures with labyrinth-like concave-convex microstructures and dendritic Ag nanostructures are obtained by immersing the etched sample into AgNO_3_ solution for a deposition process. The resultant structures with excellent chemical stability and mechanical durability can remedy the hydrophobic and destructible limitations of single bare, microstructured or nanostructured structures on Al alloys. Meanwhile, they can help to resist the seawater penetration by greatly reducing the interface interaction between corrosive ions and superamphiphobic surface owing to the newly presented solid-air-liquid contacting interface. Hence, the corrosion induced by bimetallic galvanic couple of Ag/Al can be effectively avoided, which is also found in a report by Zhang *et al.*^41^. Finally, from a perspective of the artificial neural network (ANN), a prediction model is built to optimize the cause-and-effect relationship between preparation conditions and anticorrosion parameters.

## Results and Discussion

### Preparation of hierarchical micro/nanostructures

**Figure 1:**
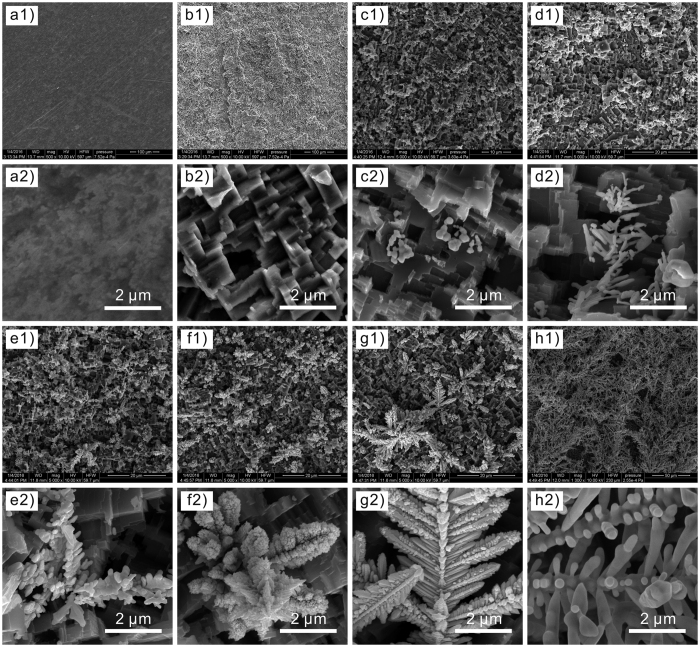
Low and high-magnified SEM images of various Al alloys surfaces. (**a**) Polished surface. (**b**) Etched surface with 4 M HCl for 18 min. (**c–h**) Etched surfaces after deposition in 30 mM AgNO_3_ solution at −0.50 V for 10, 20, 40, 60, 80, and 120 s, respectively.

Figure 1 exhibits SEM images with different magnifications of Al alloys surfaces prepared with various steps. For a polished sample, relatively smooth morphologies are shown in Fig. 1a. After the etching process, the labyrinth-like concave-convex microstructures with the ridge size of about 1.5 μm are noticed in Fig. 1b. Actually, there are many dislocations and line defects in crystal Al alloys. These high-energy defects can be preferentially dissolved in etchant than other sites^42^. Due to such selective etching effect, the resultant roughly concave-convex microstructures gradually form. With the subsequent deposition process of the etched sample in 30 mM AgNO_3_ solution at −0.50 V, dendritic Ag nanostructures can be achieved. The growth process of dendrites on etched microstructures greatly lies on the deposition time, as shown in Fig. 1c–h. With the deposition time of 10 s, a spot of nucleation sites with buds nanoparticles about 300 nm are seen in Fig. 1c due to the reduction reaction. With the deposition time extending to 20 and 40 s, Ag buds tips with low energy can promote more particles attachment leading to aggregated nuclei to form trunks and branches structures in Fig. 1d,e, respectively. Figure 1f shows the symmetrical dendritic nanostructures with side branches coupling with the trunks and branches growth due to more particles aggregations at a longer time of 60 s. Further prolonging the time to 80 and 120 s, rapidly growth of dendrites gradually evolve and then cover the etched surface in Fig. 1g,h. The schematic diagram depicting the formation process of dendrites on etched surfaces including initial reduction, nucleation, adsorption, trunks, and branches growth is illustrated in Supplementary Fig. S1. Meanwhile, such growth process is also simulated in Supplementary Video S1 according to the diffusion-limited aggregation model. In the model, a particle generating successively far from the seed just diffuses at a random path and then aggregates with the seed to form grains and dendritic structures^43^. Furthermore, the time-dependent growth from Ag buds to dendrites is also found for the gradually increased AgNO_3_ concentrations in Fig. S2 and negatively shifted potentials in Fig. S3, which is similar to the report of Radmilovic *et al.*^44^. Hence, the hierarchical structures with labyrinth-like microstructures and self-similar (proved in Fig. S4) dendritic nanostructures are achieved on Al alloy surface by ED-process steps.

### Superhydrophobicity characterization

**Figure 2:**
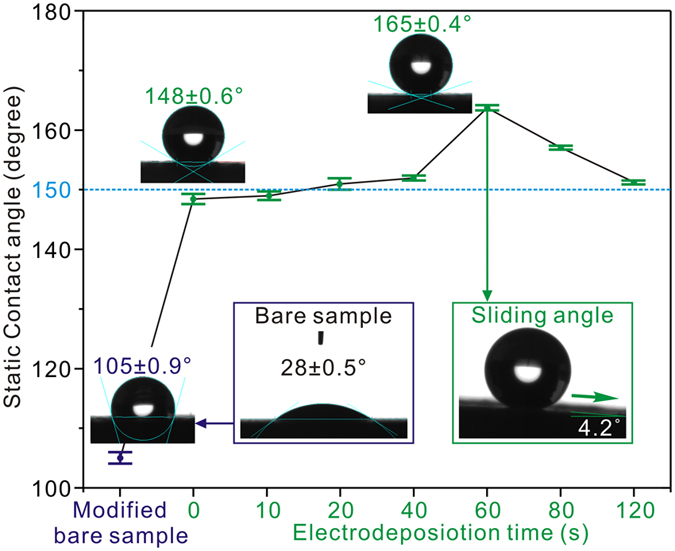
Static contact angles of water droplets (5 μL) on bare, M, EM, and EDM-process surfaces with different deposition time (deposition time of 0 s referring to the EM-process surface). The snapshot at bottom-right corner showing the sliding angle of a water droplet (5 μL) on EDM-process surface with deposition time of 60 s.

The wettabilities of Al alloys surfaces with different processes have been further explored by CAs of water droplets (5 μL) in Fig. 2. Clearly, the bare surface exhibits a hydrophilic CA of about 28 ± 0.5°. After modification, a largely increased CA of about 105 ± 0.9° is observed for the M-process surface. CAs are also investigated on EDM-process surfaces with the time-dependent deposition of etched samples in AgNO_3_ solution. For an EM-process surface (referring to the EDM-process sample with deposition time of 0 s in Fig. 2) with single microstructures, a hydrophobic CA of 148 ± 0.6° is achieved. When the deposition time increases from 10 s for Ag buds to 60 s for dendritic nanostructures, CAs for EDM-process surfaces gradually increase from 149 ± 0.8° to 165 ± 0.4° with a sliding angle of 4.2°. However, with the time extending to 80 and 120 s, the decreased CAs of 157 ± 0.3° and 151 ± 0.3° are noticed, which might result from the destructive hierarchical structures induced by excessive coverage of dendrites on etched microstructures. Hence, the wettabilities can be designed from hydrophilicity to superhydrophobicity by depositing dendritic nanostructures on etched Al alloys surfaces.

To further verify the wetting advantages of as-prepared hierarchical micro/nanostructures, a comparative wettability on DM-process surface with single nanostructures is investigated by putting a bare Al plate into 30 mM AgNO_3_ solution for 60 s at −0.50 V, as shown in Fig. S5. Obviously, the dendritic nanostructures are quickly deposited on bare surface with a hydrophobic CA of 145 ± 0.9°. By comparing the CAs on EM (148 ± 0.6°), DM (145 ± 0.9°), and EDM-process Al alloys surfaces (165 ± 0.4°), it is concluded that the hierarchical micro/nanostructures can gain the optimal wettability than single structures.

**Figure 3:**
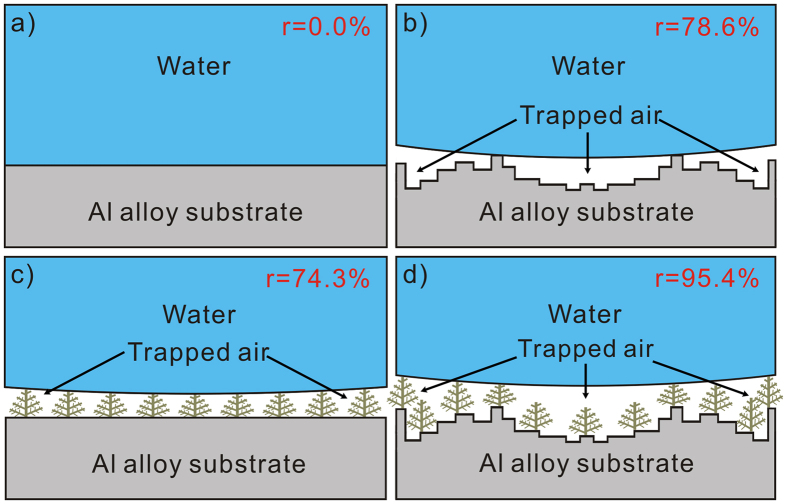
Schematic models of the contacting interface between deionized water, trapped air and (**a**) bare, (**b**) EM, (**c**) DM, and (**d**) EDM-process Al alloys surfaces.

In fact, the typical surfaces prepared with various wettabilities might result from the trapped air in corresponding structures. Figure 3 shows the contacting interfaces schematics between deionized water, air, and Al alloys surfaces with different processes. Clearly, water can completely spread out on the whole bare surface indicating a solid-liquid interface in Fig. 3a. While, for EM (Fig. 3b), DM (Fig. 3c), and EDM-process surfaces (Fig. 3d), the modified rough structures can trap air resulting in a composite solid-air-liquid interface. The effect of such composite interface on wettability can be illustrated in Cassie-Baxter equation^45^:


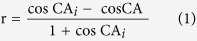


where r represents the contacting fraction with air for a droplet and CA_i_ refers to the intrinsic contact angle of 105 ± 0.9°. The equation indicates that for the EDM-process surface, the highest CA of 165 ± 0.4° implies a droplet 95.4% in contact with air and the remaining 4.6% with sample, which looks like a sphere standing on the surface. While, for EM and DM-process surfaces, the decreased r are calculated with 78.6% and 74.3%, respectively. All these suggest that the most air is trapped in EDM-process micro/nanostructures, followed by EM-process microstructures and then DM-process nanostructures, which accounts for the corresponding water-repellence abilities.

Furthermore, the comparative XPS spectra in Fig. S6 indicate that the fluoroalkyl-silane films have been self-assembled on the ED-process surface after modification. Owing to the C-H groups in modified films, a good ability of anti-OH bonds is attained suggesting excellent water-repellence ability. Meanwhile, the incorporation of low-surface-energy fluoro groups, such as C-F bonds, can further improve the wettability of the EDM-process surface.

### Superoleophobicity characterization

**Figure 4:**
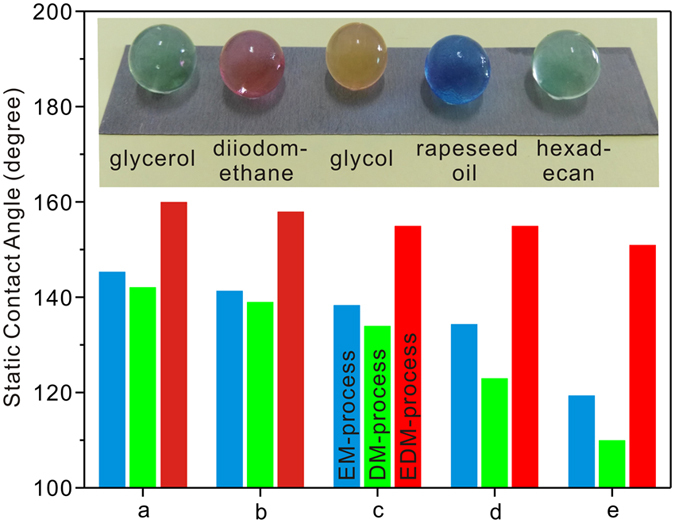
Static contact angles of (**a**) glycerol, (**b**) diiodomethane, (**c**) glycol, (**d**) rapeseed oil, and (**e**) hexadecane droplets (5 μL) on EM, DM, and EDM-process Al alloys surfaces. The insert showing the optical image of diverse oil droplets (25 μL) on EDM-process surface.

In contrast to the superhydrophobicity, preparing a superoleophobic surface is more difficult for that it has to repel oil liquids with lower surface tensions. Figure 4 shows the wettabilities on EM, DM, and EDM-process surfaces characterized by the commonly used organic solvents of glycerol (63.3 mN.m^−1^), diiodomethane (50.0 mN.m^−1^), glycol (46.5 mN.m^−1^), rapeseed oil (35.0 mN.m^−1^), and hexadecane (27.2 mN.m^−1^) in industry. It is seen that all the surfaces exhibit diverse oleophobicities. The CAs of all oil droplets (5 μL) on EDM-process surface are higher than those of EM and DM-process surfaces suggesting an enhanced oleophobicity for the hierarchical micro/nanostructures. Tuteja *et al.* have reported that preparing overhanging geometries and reentrant curvature structures can bring about excellent oleophobicity^46^. Such features can promote the formation of solid-air-oil interface and resist the penetration of corrosive oil droplets. In this work, the microstructured or nanostructured surfaces are not rough enough to achieve superoleophobicity. While for the EDM-process surface, all the oil droplets obtain the CAs higher than 150° indicating that the resultant structures exactly provide the particular features for excellent superoleophobicity. The insert in Fig. 4 refers to the optical image of glycerol, diiodomethane, glycol, rapeseed oil, and hexadecane droplets (25 μL) on the EDM-process surface. Obviously, all droplets display approximately spherical shapes standing on the as-prepared surface suggesting superior wettabilities. Furthermore, extremely low adhesion between the hexadecane and EDM-process surface is also confirmed in Fig. S7. Hence, the superamphiphobic Al alloys surface has been achieved with the EDM-process steps in this work.

### Corrosion resistances measurements

**Figure 5:**
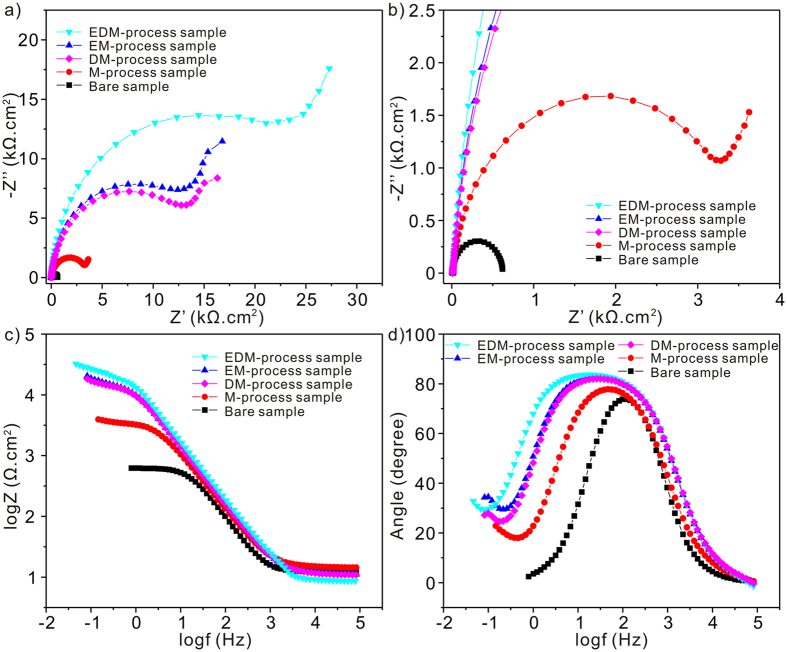
Electrochemical impedance spectroscopy of various Al alloys surfaces in 3.5 wt.% NaCl solution. (**a**) Nyquist plots. (**b**) Partial enlarged Nyquist plots. (**c**) Bode plots with impedance modules. (**d**) Bode plots with phase angles.

Corrosion resistances of bare, M, EM, DM, and EDM-process Al alloys samples in seawater are tested in Fig. 5. Figure 5a,b show the Nyquist plots measurements for various samples. Clearly, a capacitive arc relating to the charge transfer capacity at substrate/electrolyte interface is seen for all samples. A large arc size signifies improved electron transfer and corrosion resistances^47^. As shown, after the fluoroalkyl-silane modification, a larger capacitive arc is found for the bare sample. While for the EDM-process surface, it achieves the largest arc size suggesting the best corrosion resistance, followed by EM, DM, and M-process surfaces. Due to the trapped air, the EDM-process micro/nanostructures with the best liquids-repellence ability can certainly exhibit the best anticorrosion ability. Furthermore, the varied impedance modules plots for all samples are shown in Fig. 5c. A close inspection of data reveals that the impedance module at low frequency for EDM-process surface is about 2.3 × 10^4^ Ω.cm^2^ higher than EM (about 1.3 × 10^4^ Ω.cm^2^), DM (about 1.2 × 10^4^ Ω.cm^2^), M (about 3 × 10^3^ Ω.cm^2^), and bare surfaces (about 6 × 10^2^ Ω.cm^2^). The above modules values can also confirm the best anticorrosion ability for the EDM-process sample. Figure 5d exhibits the phase angles plots for various samples. Only one time constant at about 100 HZ concerning the electric double layer capacitance at substrate/electrolyte interface^48^ is found for the bare sample. While two time constants at high frequency (about 10–50 HZ) and low frequency (<0.1 HZ) are clearly seen for other modified surfaces. It is noteworthy that the time constants at low frequency for all modified samples are lower than the bare sample suggesting enhanced isolation effects to seawater for modified samples^48^.

**Figure 6:**
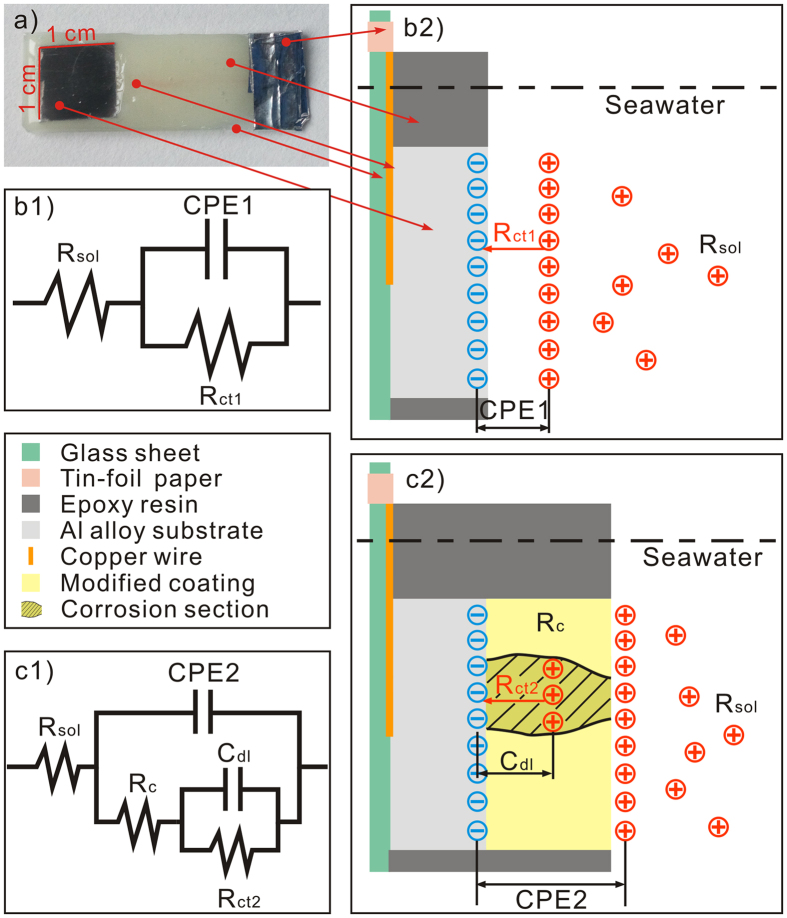
(**a**) The working electrode prepared by epoxy resin encapsulation with exposing area of 1 cm^2^ on glass sheet. Equivalent circuit patterns and corrosion mechanisms for (**b**) bare sample and (**c**) modified samples.

To accurately determine the electrochemical impedance data for bare and modified samples, the corresponding equivalent circuit patterns in seawater are fitted with ZSimDemo software in Fig. 6. Figure 6a shows the working electrode prepared by epoxy resin encapsulation with an exposing area of 1 cm^2^ for electrochemical test. The experimental EIS data for the bare sample are well fitted by the equivalent circuit pattern in Fig. 6b1. In this pattern, R_sol_, CPE1, and R_ct1_ respectively denotes the electrolyte resistance, electric double layer capacitance, and charge transfer resistance, as distinctly depicted in the substrate/electrolyte interface diagram of Fig. 6b2. Meanwhile, the equivalent circuit pattern in Fig. 6c1 can well fit the modified films. Among them, R_sol_, R_c_, CPE2, C_dl_, and R_ct2_ respectively represents the electrolyte resistance, coating resistance, coating capacitance, double layer capacitance and charge transfer resistance at the substrate/coating interface in corrosion regions, as shown in the interface corrosion diagram of Fig. 6b2. After data fittings, the calculated values of the individual electrical components are listed in Table 1. An increasing R_ct_’ (R_ct_’ = R_ct_ + R_c_) is observed for bare, M, DM, EM, and EDM-process surfaces suggesting gradually enhanced charge transfer and corrosion resistances, which coincides well with the practical EIS results.

In fact, even for a sealed modification coating, there must be some millipores allowing the corrosive ions penetration to form tiny corrosion regions, as shown in Fig. 6c2. Herein, the total corroded area fraction induced by millipores in modified film is set as α and β denotes the remaining coverage rate of the coating (α + β = 1). The relationship between α and R_ct_ is described as follows^48^:


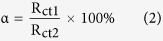


According to this equation, the coating coverage β for various samples are calculated in Table 1. Obviously, a gradually increased β is observed for the M, DM, EM, and EDM-process samples. And for the EDM-process sample, the maximal β (97.1%) suggests the highest coating coverage and the best corrosion resistance. Meanwhile, the electrochemical fitting plots presented in Fig. S8 can well match the experimental EIS results, which can further confirm the reasonability of the corrosive data simulated in this work. Besides, the potentiodynamic polarization curves in Fig. S9 are also measured to evaluate the corrosion resistances in seawater. Generally, a positive-shifting corrosion potential (E_corr_) and a lower corrosion current density (I_corr_) can be found with excellent corrosion resistance^49^. As shown, E_corr_ shifts positively from −1.300, −1.188, −0.980, −0.841, to −0.705 V for the bare, M, DM, EM, and EDM-process surfaces, respectively. Meanwhile, a gradually decreased I_corr_ for above processed surfaces is obtained respectively with 157.7, 15.64, 1.411, 0.954, and 0.094 μA.cm^−2^. Such data about anticorrosion abilities for various samples also exactly meet the EIS results. In all, the corrosion resistance in seawater can be vastly improved for the EDM-process hierarchical micro/nanostructures. Such structures can help to repel liquids and resist the seawater penetration by greatly reducing the interface interaction between corrosive ions and superamphiphobic surface owing to the newly presented solid-air-liquid contacting interface. Furthermore, the anticorrosion ability of the EDM-process surface is also found superior than some works with different preparation methods after the comparison of the corrosion current densities in Supplementary Table S1.

The chemical stability and mechanical durability of the superamphiphobic EDM-process surface are also investigated in Fig. S10. It clearly depicts a relatively stable wettability in corrosive mediums with varying pH from 1 to 14, suggesting a stable surface against chemical corrosion. The longstanding stability and durability of the superamphiphobic surface in seawater is also confirmed by characterizations of contact angles, polarization curves, and SEM images of as-prepared surfaces. Besides, superior mechanical stability of the superamphiphobic surface is evaluated in ultrasonic vibration. All these indicate that the EDM-process surface can achieve excellent chemical stability and mechanical durability for applications.

### ANN model for predicting anticorrosion property

As described above, the deposited dendrites are necessary to prepare the multifunctional Al alloys surfaces. To further investigate the relationship between the influential input variables of deposition conditions (deposition time, potentials and concentrations of AgNO_3_ solution) and the target output variable of anticorrosion characterization (R_ct_’) for EDM-process surface, a prediction model is built by a two-layer perceptron artificial neural network, as shown in Fig. S11. 90 sets of experimental data with deposition conditions and the corresponding R_ct_’ are achieved. Among them, 72 sets are used as training specimen and the remaining as testing specimen. To design the network architecture and accurately predict the input-output variables, the neuron number and training cycle number have been optimized in Figs S12 and S13, respectively. And the resultant optimal weights and biases for the trained network are listed in Table S2. Furthermore, the experimental data of the testing specimen and the predicted values attained via the trained network are exhibited in Fig. S14. After the linear fitting of the model outputs, the predicted R_ct_’ can well match the experimental data suggesting excellent fitting effect of the designed network model.

**Figure 7:**
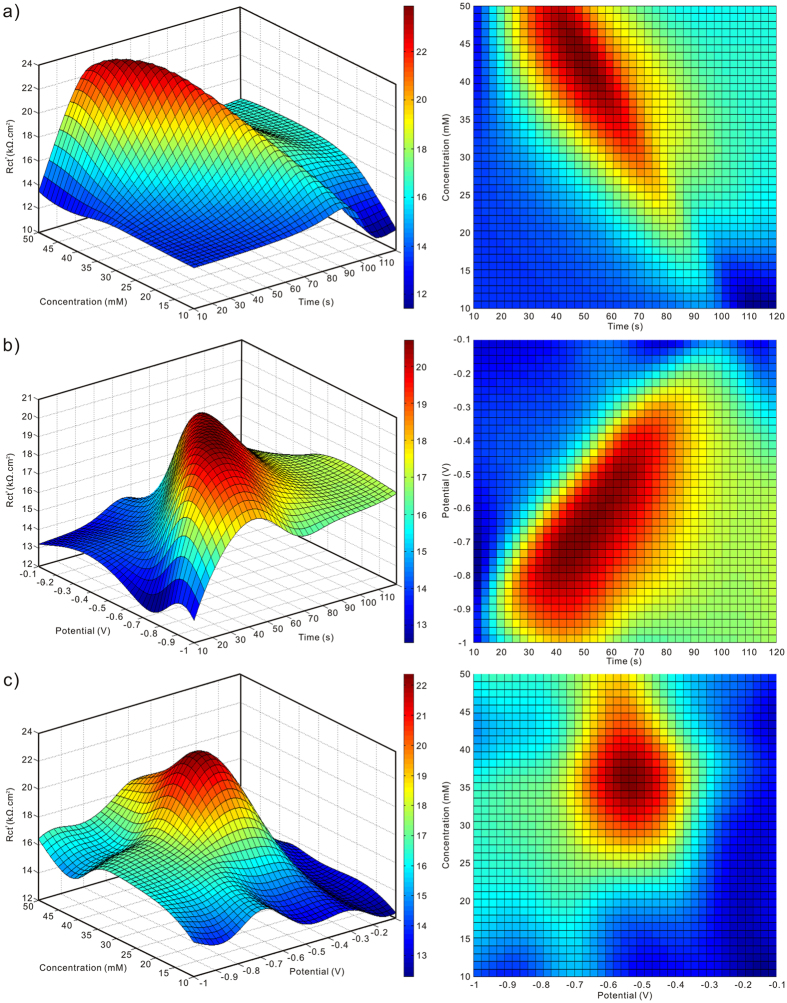
Predicted R_ct_’ for the EDM-process surface as a function of (**a**) deposition time and AgNO_3_ concentrations, (**b**) deposition time and potentials, and (**c**) deposition potentials and AgNO_3_ concentrations.

To illustrate the predicted results precisely, some three-dimensional images depicting the predicted R_ct_’ versus two deposition conditions are presented in Fig. 7. The effect of deposition time on R_ct_’ is investigated in Fig. 7a,b. Clearly, a gradually increased R_ct_’ is observed over deposition time until the maximal value appears at about 40–60 s. However, a decreasing R_ct_’ is noticed with further prolonging the deposition time. A similar phenomenon is observed in Fig. 7a,c for the concentration of AgNO_3_ solution with the optimal range of about 35–45 mM. It is also seen that the effect of deposition potentials and concentrations on R_ct_’ at lower and higher deposition time is inconspicuous. This can be respectively attributed to the incomplete deposition of Ag buds and excessive coverage of Ag dendrites on etched structures. Figure 7b,c show the effect of deposition potentials on R_ct_’. Clearly, the optimal anticorrosion behavior is seen at a negative potential of about −0.7–−0.5 V. The negative-shifting potential makes the driving force of crystallization more adequate, which can contribute to dendrites formation and result in a higher R_ct_’. However, more negative potential at about −1 V results in an enhanced corrosion rate due to the destructive hierarchical micro/nanostructures and solid-air-liquid interface induced by the excessive coverage of Ag dendrites on etched structures. In light of the model prediction in Fig. 7, the optimal deposition time from 40 to 60 s, concentration of AgNO_3_ solution from 35 to 45 mM, deposition potential from −0.70 to −0.50 V are defined, which shows good correlations with the experimental data. Hence, the established two-layer perceptron artificial neural network can be used to predict anticorrosion behaviors and optimize the influential variables of deposition conditions for prepared surfaces.

## Conclusion

A simple, low-cost, stable, and repeatable method is developed to prepare superamphiphobic and anticorrosion Al alloys surfaces. The resultant labyrinth-like microstructures and dendritic nanostructures are achieved by etch, deposition, and fluoroalkyl-silane modification. The structures can make up for the hydrophobic and destructible limitations of single bare, microstructured, and nanostructured structures for enhanced superamphiphobicity, excellent chemical stability and mechanical durability. Meanwhile, they can also resist the bimetallic galvanic corrosion by reducing interface interactions between corrosive ions and superamphiphobic surface owing to the newly presented solid-air-liquid contacting interface. Furthermore, an ANN prediction model is built to achieve the best corrosion resistance with the optimized deposition time from 40 to 60 s, AgNO_3_ concentration from 35 to 45 mM and deposition potential from −0.70 to −0.50 V.

## Methods

### Materials

Al alloys samples with dimensions of 10 × 10 × 2 mm^3^ mainly contained 95.7 wt% Al and 3.1 wt% Mg. The fluoroalkyl-silane modifier of 1H,1H,2H,2H-Perfluorodecyltrichlorosilane was obtained from Shanghai Aladdin Biological Technology Co., Ltd. Other analytical grade chemicals were purchased from Sinopharm Chemical Reagent Co., Ltd.

### Preparation of hierarchical micro/nanostructures

The samples were firstly ground by W40, W20, W10, and W3.5 sand papers, ultrasonically cleaned in deionized water, acetone, and ethanol before the nitrogen drying. Then, etching process (E-process) was conducted to attain labyrinth-like microstructures by putting cleaned samples into 4 M HCl. After etching for 18 min, samples were immediately rinsed with deionized water to stop reactions before the further drying process. Thirdly, potentiostatic deposition process (D-process) was proceeded to achieve dendritic nanostructures by putting etched samples into 30 mM AgNO_3_ solution at −0.50 V for 60 s. The electrodeposition was carried out by an electrochemical workstation (CHI660D) with a standard three-electrode system including a Pt counter electrode, a saturated calomel electrode (SCE) and a working electrode. The working electrode with an exposing area of 1 cm^2^ on glass sheet was prepared by epoxy resin encapsulation. During the electrode preparation, cracks and leakage must be avoided to ensure the fully capsulated surface. Finally, surface modification (M-process) was conducted by immersing the ED-process sample into ethanol solution of 20 mM fluoroalkyl-silane for 12 h before the further drying process at 100 °C for 2 h. All preparations were processed at room temperature of about 5 °C.

### Characterization

The morphologies were characterized by Environmental Scanning Electron Microscopy (FESEM, FEI Quanta200 FEG). The chemical states of elements were analyzed by X-ray Photoelectron Spectroscopy (XPS, VG ESCALAB MKII). The static contact angles (CAs) for water and oil droplets (5 μL) were measured by using an OCA20 system (Dataphysics GmbH, Germany). Each surface was measured for ten times to attain the average CA value. The corrosion resistance in simulated seawater (3.5 wt.% NaCl solution) was tested by using an electrochemical workstation (CHI660D). The electrochemical impedance spectroscopies (EIS) measurements were performed at open circuit potential (OCP) between 10 mHz and 100 kHz with a sinewave amplitude of 10 mV. The potentiodynamic polarization curves were recorded by linear sweep voltammetry from −1800 to −200 mV versus SCE with a scan rate of 1 mV.s^−1^. To achieve repeatable and stable results, the electrochemical tests were conducted at night to avoid ambient interferences. Before the tests, 1 h of immersion time for working electrode into electrolyte was ensured at OCP for a steady state. Such OCP variations as a function of time in 3.5 wt.% NaCl solution for various Al alloys samples were measured in Fig. S15. All the electrochemical tests were performed for four times to attain the average result.

## Additional Information

**How to cite this article**: Li, X. *et al.* Multifunctional substrate of Al alloy based on general hierarchical micro/nanostructures: superamphiphobicity and enhanced corrosion resistance. *Sci. Rep.* **6**, 35940; doi: 10.1038/srep35940 (2016).

## Supplementary Material

Supplementary Information

Supplementary Video S1

## Figures and Tables

**Table 1:** The equivalent circrsuit components parameters after the electrochemical fittings for various Al alloys surfaces.

Surfaces	R_sol_ (Ω.cm^2^)	R_ct_ (Ω.cm^2^)	CPE (μF.cm^−2^)	R_c_ (Ω.cm^2^)	C_dl_ (μF.cm^−2^)	β (%)
bare	8.33	714	56.40	—	—	—
M	11.89	3213	248.99	964	24.27	77.8
DM	11.23	12560	262.8	1165	14.0	94.3
EM	9.01	13458	353.37	2186	48.84	94.7
EDM	10.78	24859	257.41	1950	18.92	97.1
