# Supplementary material for: Multifunctional substrate of Al alloy based on general hierarchical micro/nanostructures: superamphiphobicity and enhanced corrosion resistance
Source: Sci Rep. 2016 Oct 24;6:35940. doi: 10.1038/srep35940 (PMC5075920; doi:10.1038/srep35940)
Supplement: Supplementary Information [file srep35940-s1.pdf]

Supporting Information online

Multifunctional substrate of Al alloy based on general hierarchical micro/nanostructures: superamphiphobicity and enhanced corrosion resistance

Xuewu Li[1,+], Tian Shi[2,+], Cong Liu[1], Qiaoxin Zhang[1,]* & Xingjiu Huang[1,]*

[1] School of Mechanical and Electronic Engineering, Wuhan University of Technology, 122 Luoshi Road, Wuhan 430070, P.R. China.

[2] School of Machinery and Automation, Wuhan University of Science and Technology, 947 Peace Avenue, Wuhan 430081, P.R. China.

*Correspondence and requests for materials should be addressed to Q.Z. (email: zhangqx@whut.edu.cn) or X.H. (email: xingjiuhuang@iim.ac.cn).

[+]These authors contributed equally to this work.

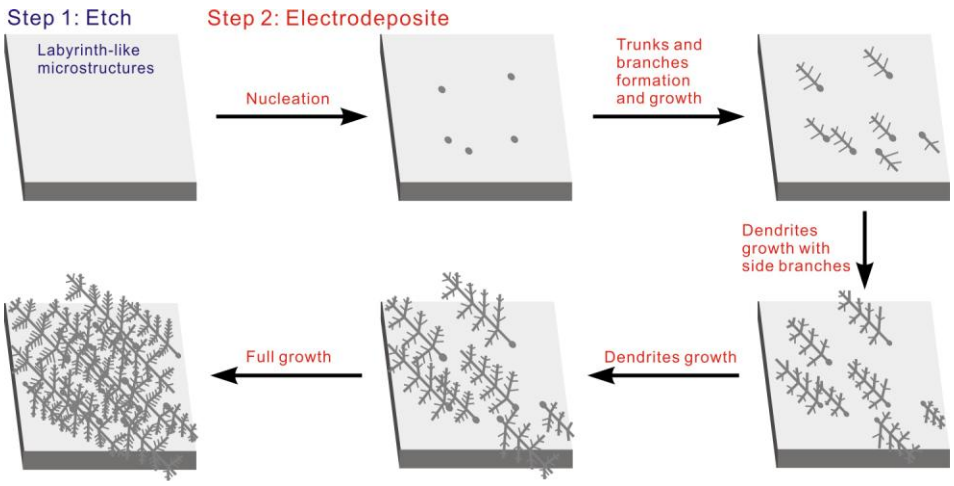

Figure S1. The schematic diagram of the growth process over deposition time from the initial Ag buds to dendrites on etched Al alloys surfaces.

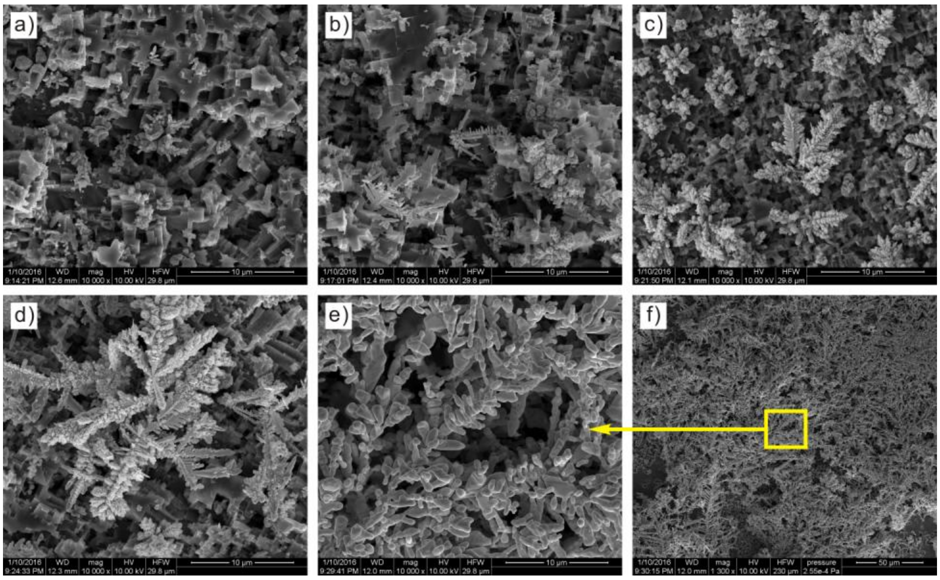

Figure S2. SEM images of the etched Al alloys surfaces after deposition at -0.50 V for 60 s in various $AgNO_3$ solution concentrations of (a) 10, (b) 20, (c) 30, (d) 40, and (e) 50 mM. (f) Low-magnified SEM image of (e).

The effect of $AgNO_3$ solution concentrations on dendrites formation is investigated on etched Al alloys surfaces in **Fig. S2**. When the concentrations increase from 10, 20, 30, 40 to 50 mM at a constant potential of -0.50 V for 60 s, the time-dependent growth process from buds nanoparticles to fully grown dendrites in Fig. 1 is similar to this concentration-dependent process, which can also be found in the report by Radmilovic *et al*[1]. Actually, the increased concentrations can promote the reduction reaction ($Ag^+ + e^- \rightarrow Ag$) resulting in more abundant aggregation and growth from nanoparticles to dendritic structures.

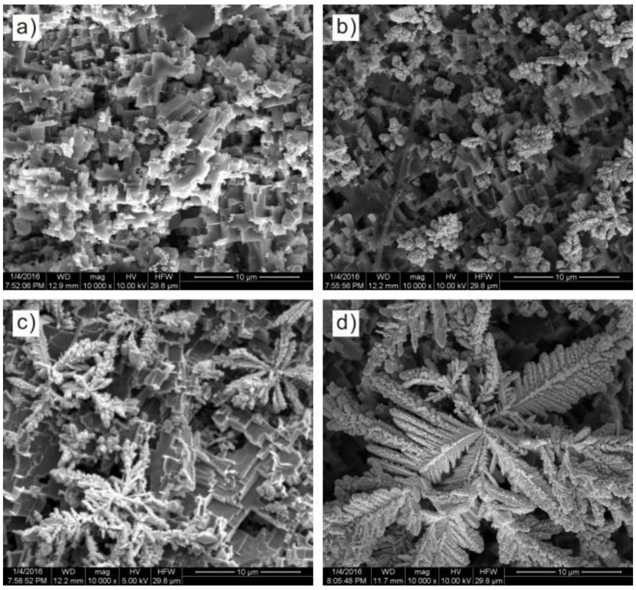

Figure S3. SEM images of the etched Al alloys surfaces after deposition in 30 mM $AgNO_3$ solution for 60 s at various potentials of (a) -0.30, (b) -0.50, (c) -0.70, and (d) -1.00 V.

Fig. S3 exhibits the effect of deposition potentials on dendrites formation on the etched Al alloys surfaces. As the potentials negatively shift from -0.30, -0.50, -0.70 to -1.00 V in constant 30 mM $AgNO_3$ solution for 60 s, the analogously time-dependent growth process from Ag buds to fully grown dendrites can also be found. Actually, when the deposition potential shifts negatively, an enhanced crystallization driving force is attained, which can promote more Ag^+ diffusion to form dendrites.

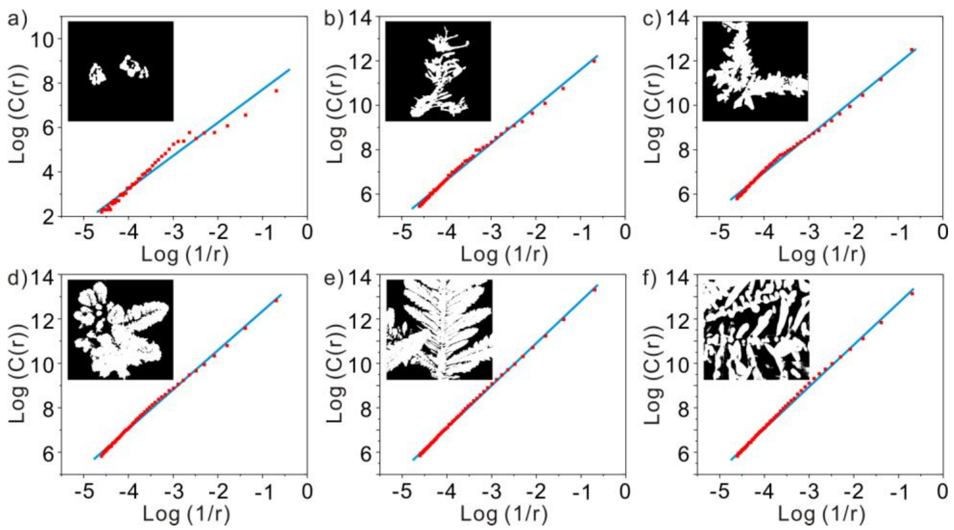

Figure S4. Fractal dimensions of Ag patterns at different deposition time of (a) 10, (b) 20, (c) 40, (d) 60, (e) 80, and (f) 120 s. The inserts referring to the binary images obtained by im2bw function in MATLAB.

To determine the deposited Ag patterns, a box counting algorithm[2] is used to calculate the fractal dimensions (D) of time-dependent dendritic structures on etched surfaces. Before the calculation, the binary images of the deposited structures are obtained in **Fig. S4** using an im2bw function in MATLAB via the threshold transformation of the corresponding SEM images in Fig. 1c2-h2. In the box counting method, squared-grid patterns with various sizes (r) are set to cover the whole binary image. The relationship between the amount of the non-vacuous grids (C(r)), r, and D can be statistically described as: $C(r) \sim r^{-D}$. **Fig. S4** also shows the plots of logC(r) vs. $\log r^{-1}$, which are linearly fitted with diverse slopes namely fractal dimensions of Ag patterns. It can be seen that the time-dependent D linearly increases from 1.52 for Ag buds to 1.87 for fully dendrites. These results coincide well with the reported dimensions for self-similar dendritic structures of about 1.50-1.70[3], 1.83[2] and 1.70[4]. Therefore, the dendrites prepared in this work can exhibit self-similar patterns.

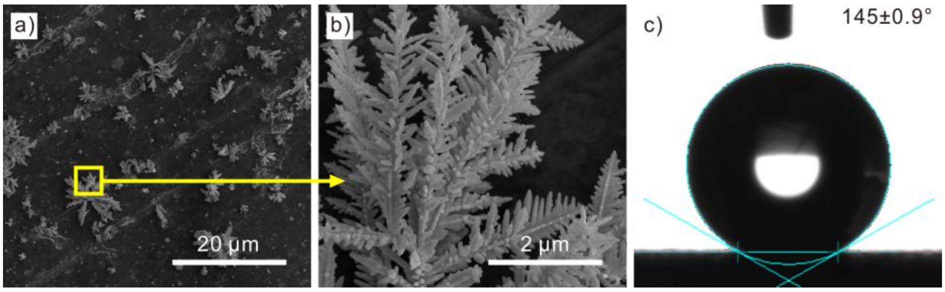

Figure S5. (a) Low and (b) high-magnified SEM images of the deposited Al alloy surface by putting a bare Al plate into 30 mM $AgNO_3$ solution for 60 s at -0.50 V. (c) Surface wettability after modification of the structures in (a).

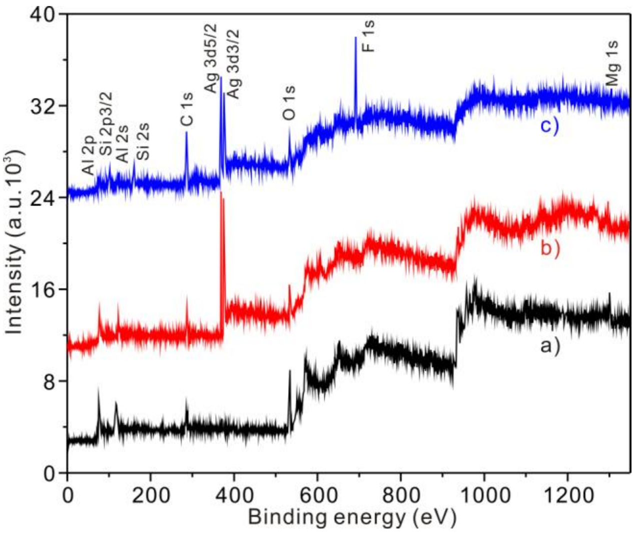

Figure S6. XPS spectra of (a) bare, (b) ED, and (c) EDM-process Al alloys surfaces.

Fig. S6 shows the comparative XPS spectra of bare (**Fig. S6a**), ED (**Fig. S6b**), and EDM-process Al alloys surfaces (**Fig. S6c**). After the bare surface prepared with ED-process steps, new element peaks of Ag 3d5/2 (368.4 eV) and Ag 3d3/2 (374.5eV) are seen indicating that the deposited silver exists with the form of metallic silver[5]. After the fluoroalkyl-silane modification of ED-process surface, new element peaks of F 1s (685.7 eV), Si 2s (150.5 eV), and Si 2p3/2 (100.1 eV) accompanying with the reduced intensity of Al 2p (75.8 eV), Al 2s (119.4 eV), Mg 1s (1304.6 eV) peaks and greatly increased intensity of C 1s (284.6 eV) peak are observed indicating that the fluoroalkyl-silane film has been self-assembled on the as-prepared surface. Owing to the C-H groups in modified films, a good ability of anti-OH bonds is attained suggesting excellent water-repellence ability. Meanwhile, the incorporation of low-surface-energy fluoro groups, such as C-F bonds, can further improve the wettability of the EDM-process surface.

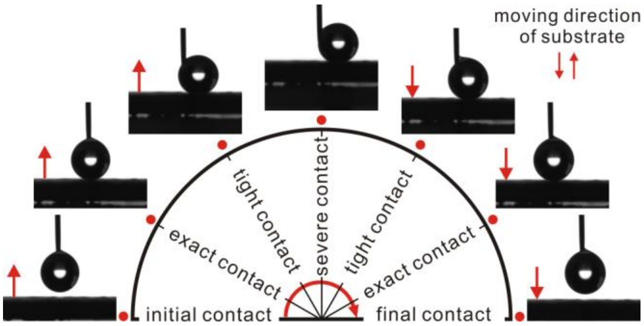

Figure S7. Sequential photos about the adhesive behavior test between the EDM-process Al alloy surface and a hexadecane droplet (5 μL) adsorbed on a fixed syringe needle by making different contact states.

A superoleophobic surface is identified not only with a CA higher than 150°, but also with low adhesion to the substrate surface[6]. **Fig. S7** depicts a test about adhesive behavior between a hexadecane droplet (5 μL) with low surface tension and the EDM-process surface. In the whole experiment, the substrate keeps towards or away from the droplet adsorbed on a fixed syringe needle. Clearly, the droplet is gradually raised with the rising substrate but without fleeing the needle. After breaking away from the substrate, the droplet still keeps contact with the needle without oil residues on substrate surface. Therefore, the adhesive performance between the oil droplet and the EDM-process surface is quietly weak and even can be ignored.

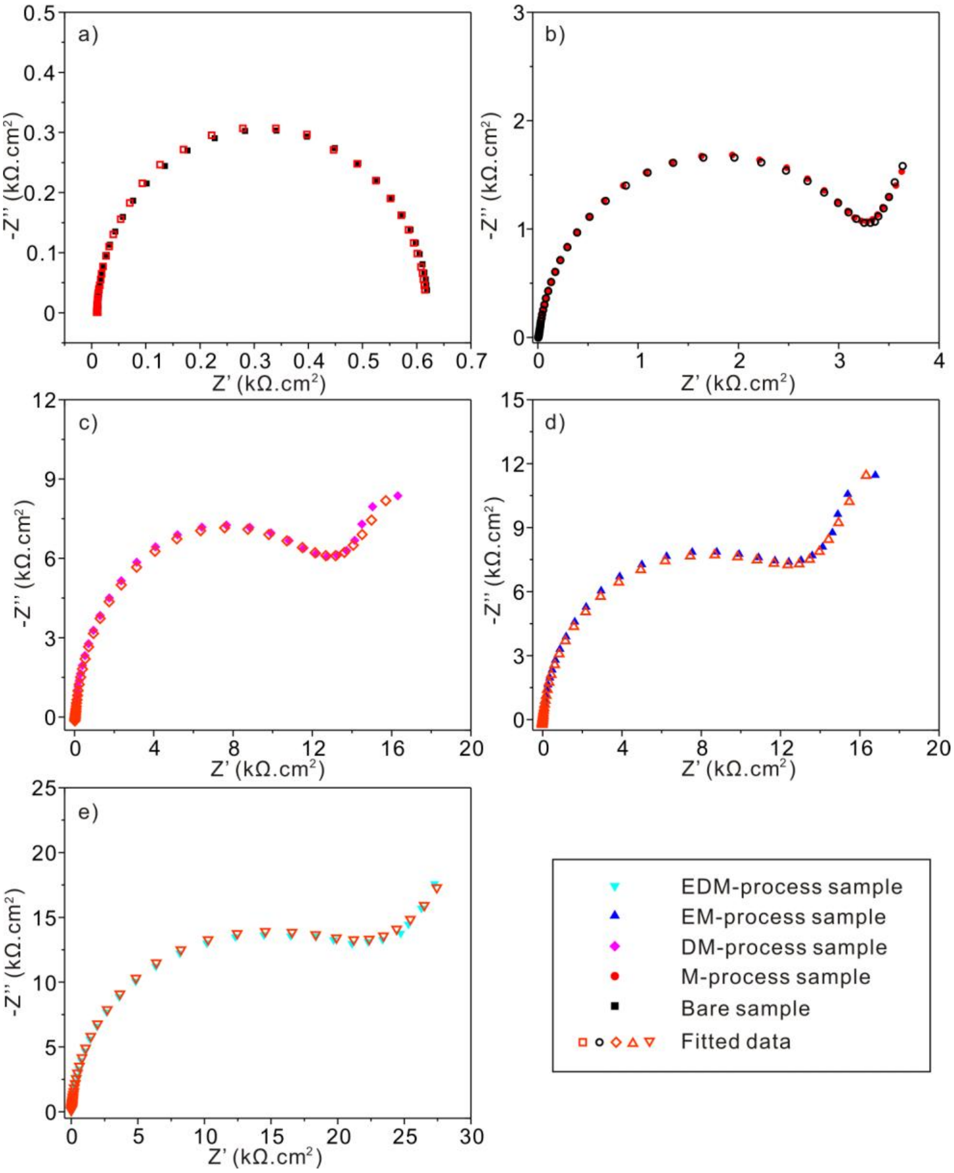

Figure S8. Nyquist plots from electrochemical impedance spectroscopy measurements and the corresponding fitting data from equivalent circuit patterns by using the ZSimDemo software for (a) bare, (b) M, (c) DM, (d) EM, and (e) EDM-process Al alloys samples.

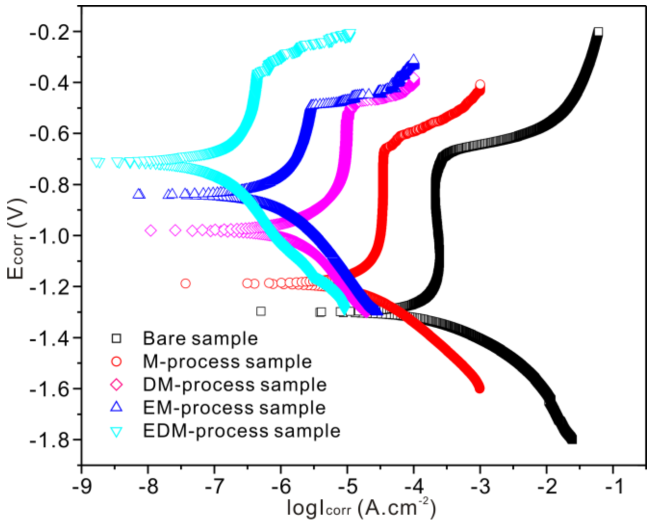

Figure S9. Potentiodynamic polarization curves of bare, M, DM, EM and EDM-process Al alloys samples in 3.5 wt.% NaCl solution.

References	Preparation conditions	Modifiers	I_{corr} (μA.cm^{-2})	Structures
7	Immersion and ultrasound	STA	1.25	Micropits with nanoparticles
8	Boiling water etch	GPS then PS-COOH	11.6	Nanopillars with hollows
9	NaOCl etch	HTS	2.95	Micropits with nanoparticles
10	NaOH and $CuCl_2$ etch then anneal	None	1.84	Microdendrites with nanoparticles
11	NaOH etch	GPTMS and MTMS crosslinked with HMMM	0.9	Microporous with craters
12	Acid etch and anodization	FDTS	0.1-1	Microconcaves with nanopores
This work	HCl etch	FDTS	0.954	Convex/concave microstructures
This work	HCl etch then electrodeposition	FDTS	0.094	Convex/concave microstructures with nanodendrities

Table S1. Comparison of the anticorrosion properties of EDM-process surface in this work and some reported works with different preparation methods. (STA: Stearic acid. GPS: 3-glycidoxypropyl trimethoxysilane. HTS: Hexadecyltrimethoxysilane. GPTMS: Glycidoxytrimethoxysilane. MTMS: Methyltrimethoxysilane. HMMM: Hexamethylmethoxymelamine. FDTS: 1H,1H,2H,2H-Perfluorodecyltrichlorosilane.)

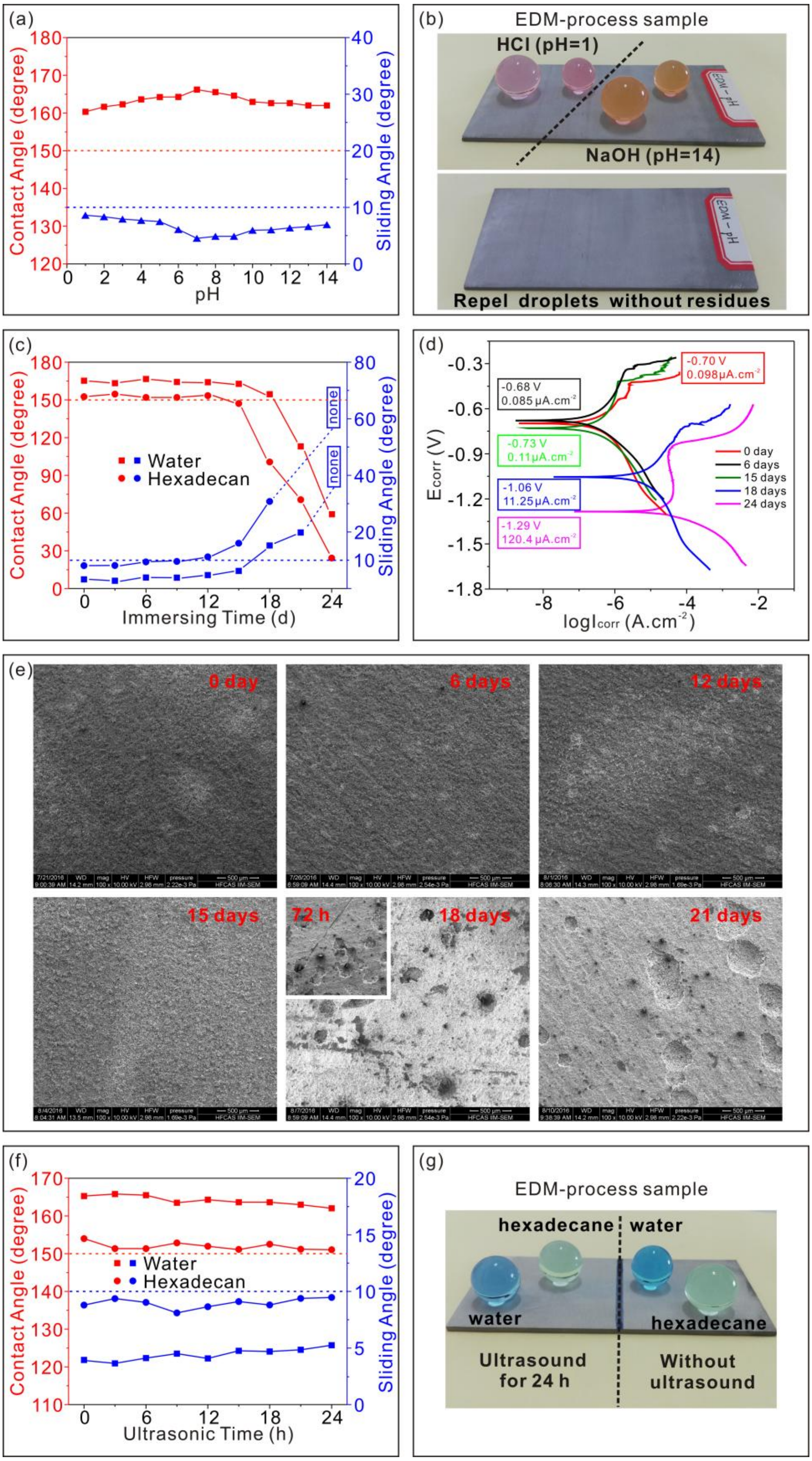

Figure S10. Liquid-repellence abilities of the superamphiphobic EDM-process surface under different conditions. (a) Effect of pH values on contact and sliding angles. (b) Optical images

of corrosive droplets on the surface. (c) Hexadecane and water contact angles, (d) potentiodynamic polarization curves, and (e) SEM images of the surfaces with different immersion time in seawater. (f) Hexadecane and water contact angles of the surfaces with different ultrasound time in water. (g) Optical images of water and hexadecane on the surface after ultrasound for 24 h. The insert in (e) showing SEM image of the bare surface after immersion into seawater for 72 h.

The chemical stability and mechanical durability of the superamphiphobic EDM-process surface are investigated in **Fig. S10**. **Fig. S10a** shows the relationship between pH values and droplets contact angles on the EDM-process surface. It clearly depicts a relatively stable wettability for corrosive mediums with varying pH from 1 to 14, suggesting a stable surface against chemical corrosion. As shown in **Fig. S10b**, the as-prepared surface can repel NaOH (pH=14) and HCl (pH=1) droplets without residues left on prepared surface by slightly slope the sample. The stability and durability of the superamphiphobic surface in 3.5 wt.% NaCl solution (pH=5.5) are also studied in **Fig. S10c**. Clearly, there are no obvious fluctuations for hexadecane and water contact angles after immersion into seawater for 15 days. After that, the wettabilities for both droplets gradually decline and the superamphiphobicity disappears. The polarization curves in **Fig. S10d** are tested to characterize anticorrosion behaviors of the EDM-process surface over immersion time in seawater. The corrosion current density can remain at about 0.1 $\mu A.cm^{-2}$ after immersion for 15 days. Then, it greatly increases to 120.4 $\mu A.cm^{-2}$ after 24 days, which is close to the bare sample of 157.7 $\mu A.cm^{-2}$ suggesting that the as-prepared protective surface has been destroyed. The above analyses can also be confirmed

by SEM images of the surfaces in **Fig. S10e**. When the samples are exposed to seawater for 0, 6, 12 and 15 days, few morphologies changes can be found indicating excellent chemical stability. However, a small quantity of pitting corrosion can be seen after 18 days, which is similar to the bare surface after immersion into seawater for 72 h in the insert of **Fig. S10e**. When the immersion time is prolonged to 21 days, the surface is seriously destroyed owing to the erosion of chloride ions, which accounts for its decreased corrosion resistance. The mechanical stability of the superamphiphobic surface is also evaluated in ultrasonic vibration. It is observed that the as-prepared surface can still exhibit excellent repellence to water and hexadecane after ultrasound for 24 h, as shown in **Fig. S10f** and **g**. All these suggest that the EDM-process surface can achieve superior chemical stability and mechanical durability for applications.

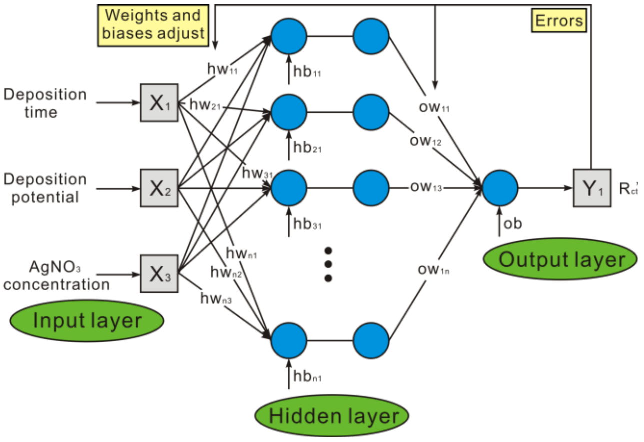

Figure S11. The architecture of a two-layer perceptron artificial neural network used to model and predict the relationship between the influential input variables of deposition conditions (deposition time, potentials and concentrations of $AgNO_3$ solution) and the target output variable of anticorrosion characterization (R_{ct}') for EDM-process surface.

Fig. S11 exhibits a two-layer perceptron artificial neural network used to model and predict the relationship between the influential input variables of deposition conditions and the target output variable of anticorrosion characterization (R_{ct}') for EDM-process surface. In the network, the nodes simulating the function of artificial neurons are connected to every other node in next layer. Meanwhile, tansig and purelin functions are used as the transfer functions of hidden layer and output layer, respectively. Before training the network with the scaled-conjugate gradient back-propagation algorithm, all data are normalized. After being trained with experimental specimen, the experimental outputs for testing specimen are compared with the corresponding model outputs to calculate the errors of output nodes. And also by adjusting weights and biases, the network is trained to decrease such errors. The learning process sustained time after time until the mean squared errors of all training specimen at output nodes converge to a permitted range.

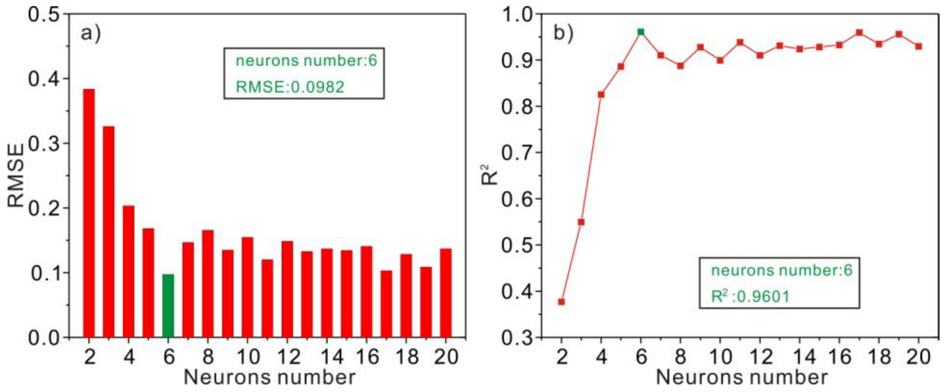

Figure S12. (a) The root mean square error (RMSE) and (b) correlation coefficient (R^2) of the model and experimental outputs versus the neurons number in hidden layer.

The two-layer perceptron artificial neural network with a hidden layer is considered in this work. To further ensure the neurons number in hidden layer, series of topological structures with the neurons number varying from 2 to 20 are used. To avoid the correlation caused by the random weights and biases initialization, every topology is repeated for 5 times. The optimal network architecture is finally determined by computing the minimum value of the root mean square error (RMSE) and the maximal correlation coefficient (R^2) of the model and experimental outputs in testing specimen, as shown in **Fig. S12**. Clearly, the hidden layer with 6 neurons is observed with the minimal RMSE of 0.0982 (**Fig. S12a**) and the maximal R^2 of 0.9601 (**Fig. S12b**). Hence, a well-defined two-layer perceptron artificial neural network with 6 neurons in hidden layer is achieved to model and predict the input-output variables.

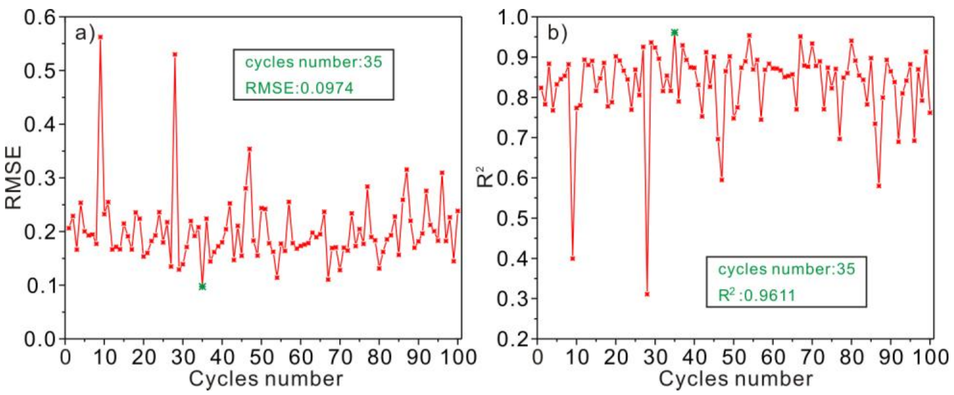

Figure S13. (a) RMSE and (b) R^2 of the model and target outputs versus the cycles number of the network.

Hidden layer				Output layer	
hw_{11} 1.2729	hw_{12} 7.0475	hw_{13} -6.7316	hb_{11} -3.3753	ow_{11} -0.0832	ob -0.8564
hw_{21} -3.6644	hw_{22} 2.8658	hw_{23} 5.5816	hb_{21} 2.1187	ow_{12} 0.6130	–
hw_{31} 4.6642	hw_{32} 1.2689	hw_{33} 0.0083	hb_{31} -2.8124	ow_{13} -0.6407	–
hw_{41} -9.4647	hw_{42} 1.4684	hw_{43} 3.7128	hb_{41} 9.0967	ow_{14} -0.4726	–
hw_{51} 2.8402	hw_{52} -0.0937	hw_{53} -2.9056	hb_{51} 0.2978	ow_{15} 0.5561	–
hw_{61} -0.8560	hw_{62} -10.5786	hw_{63} -5.0967	hb_{61} -4.9027	ow_{16} -0.2480	–

Table S2. The weights and biases in hidden and output layers after the neural network being trained for 35 cycles. (hw: weight in hidden layer, hb: bias in hidden layer, ow: weight in output layer, ob: bias in output layer)

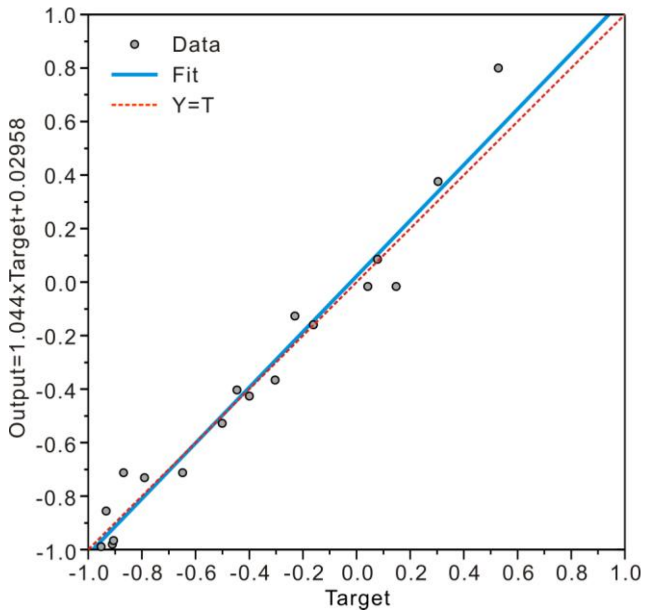

Figure S14. Regression plot between experimental R_{ct}' of the testing specimen and the predicted values attained via the trained artificial neural network.

To obtain the optimal weights and biases, the network is trained for 100 cycles and the corresponding RMSE and R^2 versus the cycle numbers are shown in **Fig. S13a** and **b**, respectively. Clearly, the minimal RMSE of 0.0974 and the maximal R^2 of 0.9611 for model and experimental data are found after the network being trained for 35 cycles and also the corresponding weights and biases are listed in **Table S2**. Furthermore, the experimental data for the testing specimen and the predicted values attained via the trained network are exhibited in **Fig. S14**. After the linear fitting of the model outputs, the predicted R_{ct}' can well match the experimental data suggesting excellent fitting effect of the optimized network model in this work.

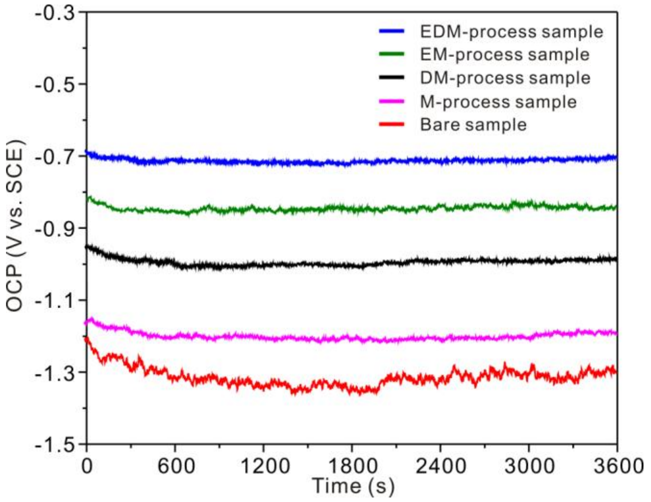

Figure S15. OCP variations as a function of time in 3.5 wt.% NaCl solution for bare, M, EM, DM, and EDM-process Al alloys samples.

Fig. S15 shows the time-dependent OCP in 3.5 wt.% NaCl solution for bare, M, EM, DM, and EDM-process Al alloys samples. It is seen that the OCP curve for bare sample slightly decreases from -1.195 V to -1.300 V within 10 minutes of immersion, and finally keeps at about -1.297 V. Such falling curve is also found with large fluctuations, which is attributed to some localized breakdowns caused by the ions attack on surfaces owing to the direct solid-liquid interface interactions. While for other modified samples, more stable OCP curves towards positive directions are found suggesting improved corrosion resistances. Due to the hydrophobic behaviors, modified surfaces can repel corrosive ions penetrations by reducing the substrate/electrolyte interactions for the newly-presented solid-air-liquid interface. Meanwhile, the slightly decreased OCP values in early stages are also seen for each modified sample, which results from the millipores in coatings allowing the corrosive ions diffusions to substrate surfaces. A close inspection of OCP data for various modified samples reveals that the EDM-process sample with the maximal coating coverage rate is certainly seen with the

best isolation effect and the highest OCP value of about -0.708 V in the stable stage, followed by EM (-0.844 V), DM (0.937 V), and M-process samples (-1.195 V). Such observations about anticorrosion behaviors are in good accordance with the later electrochemical results.

References

1. Radmilovic, V. V., Kacher, J., Ivanovic, E. R., Minor, A. M. & Radmilovic, V. R. Multiple twinning and stacking faults in silver dendrites. *Cryst. Growth Des*. **16,** 467-474 (2016).
2. Wang, Z., Zhao, Z. & Qiu, J. A general strategy for synthesis of silver dendrites bygalvanic displacement under hydrothermal conditions. *J. Phys. Chem. Solids* **69,** 1296-1300 (2008).
3. Sivasubramanian, R. & Sangaranarayanan, M. V. A facile formation of silver dendrites on indium tin oxide surfacesusing electrodeposition and amperometric sensing of hydrazine. *Sensor. Actuat. B-Chem.* **213,** 92-101 (2015).
4. Witten, T. A. & Sander, L. M. Diffusion-limited aggregation: a kinetic critical phenomenon. *Phys. Rev. Lett*. **47,** 1400-1403 (1981).
5. Stathatos, E. & Lianos, P. Photocatalytically deposited silver nanoparticles on mesoporous TiO_2 films. *Langmuir* **16,** 2398-2400 (2000).
6. Teng, C. *et al.* Stable underwater superoleophobic and low adhesive polypyrrole nanowire mesh in highly corrosive environments. *Soft Matter.* **11,** 4290-4294 (2015).
7. Rezayi, T. & Entezari, M. H. Toward a durable superhydrophobic aluminum surface by etching and ZnO nanoparticle deposition. *J. Colloid Interf. Sci.* **463,** 37-45 (2016).
8. Feng, L. B., Yan, Z. N., Qiang, X. H., Wang, Y. P. & Liu, Y. H. Polystyrene-grafted Al surface with excellent superhydrophobicity and corrosion resistance. *Surf. Interface Anal.* **47,** 506-513 (2015).
9. Lv, D. M., Ou, J. F., Xue, M. S. & Wang, F. J. Stability and corrosion resistance of

superhydrophobic surface on oxidized aluminum in NaCl aqueous solution. *Appl. Surf. Sci.* **333,** 163-169 (2015).

10. Cheng, Y. Y., Lu, S. X. & Xu, W. G. Controllable wettability of micro- and nano-dendritic structures formed on aluminum substrates. *New J. Chem.* **39,** 6602-6610 (2015).
11. Wankhede, R. G., Morey, S., Khanna, A. S. & Birbilis, N. Development of water-repellent organic-inorganic hybrid sol-gel coatings on aluminum using short chain perfluoro polymer emulsion. *Appl. Surf. Sci.* **283,** 1051-1059 (2013).
12. Liu, Y., Cao, H. j., Chen, S. G. & Wang, D. A. Ag nanoparticle-loaded hierarchical superamphiphobic surface on an Al substrate with enhanced anticorrosion and antibacterial properties. *J. Phys. Chem. C* **119,** 25449-25456 (2015).

Video Legends

Video S1. The time-dependent growth process from Ag buds to dendrites simulated via the diffusion-limited aggregation model.
